# Identification and characterization of SET domain family genes in bread wheat (*Triticum aestivum* L.)

**DOI:** 10.1038/s41598-020-71526-5

**Published:** 2020-09-03

**Authors:** Ritu Batra, Tinku Gautam, Sunita Pal, Deepti Chaturvedi, Rakhi, Irfat Jan, Harindra Singh Balyan, Pushpendra Kumar Gupta

**Affiliations:** Department of Genetics and Plant Breeding, CCS University, Meerut, Uttar Pradesh 250004 India

**Keywords:** Computational biology and bioinformatics, Plant sciences

## Abstract

SET domain genes (SDGs) that are involved in histone methylation have been examined in many plant species, but have never been examined in bread wheat; the histone methylation caused due to SDGs is associated with regulation of gene expression at the transcription level. We identified a total of 166 bread wheat TaSDGs, which carry some interesting features including the occurrence of tandem/interspersed duplications, SSRs (simple sequence repeats), transposable elements, lncRNAs and targets for miRNAs along their lengths and transcription factor binding sites (TFBS) in the promoter regions. Only 130 TaSDGs encoded proteins with complete SET domain, the remaining 36 proteins had truncated SET domain. The TaSDG encoded proteins were classified into six classes (I–V and VII). In silico expression analysis indicated relatively higher expression (FPKM > 20) of eight of the 130 TaSDGs in different tissues, and downregulation of 30 TaSDGs under heat and drought at the seedling stage. qRT-PCR was also conducted to validate the expression of seven genes at the seedling stage in pairs of contrasting genotypes in response to abiotic stresses (water and heat) and biotic stress (leaf rust). These genes were generally downregulated in response to the three stresses examined.

## Introduction

Eukaryotic DNA is packaged in the form of chromatin, which itself is organized in the form of nucleosomes. In turn, each nucleosome consists of two super-helical turns of DNA wrapped around a histone (H) octamer consisting of one H3/H4 tetramer and two H2A/H2B dimers^1^. The nucleosomes are organized into higher order structures stabilized by histone H1. It is widely known that specific amino acid residues of histone tails are post-transcriptionally modified due to acetylation, phosphorylation, methylation, ubiquitylation, and SUMOylation, although all these modifications are reversible^2,3^. Post-transcriptional methylation of specific amino-acid residues in histone proteins at specific lysine (K) residues is an epigenetic modification that regulates expression of many genes associated with these modified histones. Besides other modifications, these epigenetic modifications are mediated by proteins called histone methyltransfersaes (HMTase). A fairly large number of these proteins contain a SET domain, thus constituting a family of SET-domain methyltransferases. All HMTases belong to this family of SET domain proteins, with the solitary exception of the HMTase that is involved in methylation of H3K79^4–6^. In plants, histone methylation has been reported in lysine residues at positions 4, 9, 27, 36 and 79 of H3 and position 20 of H4^7,8^, which are all important epigenetic marks. Each of these lysine residues may carry one, two or three methyl residue(s) so that the corresponding states are described as mono-, di- and tri-methylation states. In addition to catalyzing methylation of histone proteins, SET domain proteins are also known to be involved in methylation of few other proteins including large subunit of the Rubisco holoenzyme complex^9^.

The acronym SET [**S**u(var)3–9, **E**nhancer-of-zeste and **T**rithorax] was derived from three different conserved regions identified in the following three different proteins in *Drosophila*: (i) SUPPRESSOR OF VARIEGATION 3–9 [SU(VAR)3–9], a modifier of position-effect variegation^10^; (ii) ENHANCER OF ZESTE [E(Z)], the polycomb-group chromatin regulator^11^ and (iii) TRITHORAX (TRX), the trithorax-group chromatin regulator^12,13^. The SET domain itself consists of ~ 130–150 amino acids. Some conserved residues within the SET domain sequence form a knot-like structure (catalytic core), which constitutes the site for histone methyltransferase (HMT) activity^14^; methylation occurs, when AdoMet (methyl group donor) and the substrate lysines (e.g. H3Ks) are brought into close proximity. The hydroxyl group of a highly conserved tyrosine in the catalytic core of the SET domain forms Van der Waals interactions with the ribose of AdoMet and also deprotonate the amino group of the target lysine residue^15^. This deprotonation primes the lysine in the side chain to make a nucleophilic attack on the methyl group of the AdoMet molecule, thus facilitating the transfer of methyl group to the lysine residue, resulting in the production of methylated histone and the co-factor AdoHcy (byproduct of AdoMet demethylation)^16–19^. The crystal structures of SET-domain proteins suggest that the SET domain is folded into several small β sheets^20^. Often, slight variation is caused in the conformation of SET domain due to β-sheets. Such conformational changes modify the specificity of the target residue for methylation and enable methyltransferases to target several different residues.

A SET domain is often flanked by N-terminal pre-SET and C-terminal post-SET domains. The pre-SET domain region contains nine cysteine residues that form triangular zinc clusters, which bind the zinc atoms and stabilize the structure. The C-terminal post-domain, on the other hand, has three cysteine residues which participate in the formation of a zinc-binding site. It has been shown that both N- and C-terminal regions flanking the SET-domain are also required for HMTase activity^18^. The interaction between the pre-SET domain and the catalytic center of the SET domain is important for enzyme function^16^.

The SET-domain proteins have now been found in all eukaryotes/prokaryotes except some lower algae. Among plants, these proteins have been best characterized in *Arabidopsis thaliana*. The genes encoding these proteins have been variously classified in different studies using different criteria (including the site of methylation); following are some details of four such studies involving classification of SET domain genes: (i) 37 Arabidopsis genes were placed in four classes on the basis of characteristics of SET domain, cysteine-rich region and additional conserved domains^21^; (ii) 32 Arabidopsis genes and 22 maize genes were placed in five classes (I-V), based on phylogenetic analyses and domain organization^22,23^; the genes in a particular class were further classified in one (class IV) to seven (class V) orthology groups on the basis of position of SET domain and presence of other domains, the total number of orthology groups in five classes being 19. This system of placement of genes in orthology groups within a class (for classes I to V) was followed in the present study also; (iii) 47 Arabidopsis genes, 37 rice genes and 35 maize genes were placed in seven classes, on the basis of annotation using Pfam and ChromDB database^23^; and (iv) 31 Arabidopsis genes encoding proteins with SET domain were placed in five classes^24^. On the basis of their domain architectures and/or differences in enzymatic activity, a consensus classification containing seven classes has emerged; a summary of these seven classes (along with orthology groups in each class) is presented in Table 1. Proteins within each class often share a higher level of similarity in the SET domain, relative to those from different classes. According to this classification, classes I-V have proteins with complete SET domain whereas proteins belonging to classes VI and VII have an incomplete/truncated SET domain. Members of classes I-VI are known to be involved in methylation of histone proteins, whereas members of class VII are involved in methylation of non-histone proteins. Members of individual classes of SET domain proteins have specificity to the following substrates: class I for H3K27, classes II and VI for H3K36, classes III and IV for H3K4, and class V for H3K9 (Table 1)^23^.

**Table 1:** Details of 7 classes of SET domain proteins and the corresponding genes in *Arabidopsis thaliana*.

Class of SET protein; methylation site	OG*	Genes in OG*	Domains present	Function
I. Enhancer of Zeste [E(z)] homologs; H3K27	1	*MEA* (*SDG5*)	CXC (cysteine-rich region), SET	Repress homeotic gene expression
	2	*CLF* (*SDG1*)		
	3	*SWN* (*SDG10*)		
II. ASH1 homologs and related proteins (OGs based on position of SET domain); H3K36	1	*ASHH3* (*SDG7*), ASHH4 (SDG24)	AWS, SET, Post-SET	Positive regulator of homeotic gene expression
	2	*ASHR3* (*SDG4*)	PHD, AWS, SET, Post-SET	
	3	*ASHH1* (*SDG26)*	Zf, AWS, SET, Post-SET	
	4	*ASHH2* (*SDG8*)	AWS, SET, Post-SET, CW	
III. Trithorax homologs and related proteins ; H3K4	1	*ATX1* (*SDG27*), *ATX2* (*SDG30*)	SET, Post-SET, PWWP, FYRN, FYRC, PHD	Positive regulator of homeotic gene expression
	2	*ATX3* (*SDG14*), *ATX4* (*SDG16*), *ATX5* (*SDG29*)	SET, Post-SET, PWWP, PHD	
	3	*ATXR3* (*SDG2*)	SET, Post-SET	
	4	*ATXR7* (*SDG25*)	SET, Post-SET	
IV. Proteins with a SET and a PHD domain; H3K4	1	*ATXR5* (*SDG15*), *ATXR6* (*SDG34*)	SET, PHD	Cell cycle regulation or DNA replication
V. Suppressor of variegation [Su(var)] homologs (SUVH) and relatives (SUVR); H3K9	1	*SUVH1* (*SDG32*), *SUVH3* (*SDG19*), *SUVH7* (*SDG17*), *SUVH8* (*SDG21*)	YDG, Pre-SET, SET, Post-SET	Heterochromatin formation and DNA methylation in locus specific manner
	2	*SUVH4* (*SDG33*), *SUVH6* (*SDG23*)	YDG, Pre-SET, SET, Post-SET	
	3	*SUVH2* (*SDG3*), *SUVH9* (*SDG22*)	YDG, Pre-SET, SET, Post-SET	
	4	*SUVR3* (*SDG20*)	Pre-SET, SET, Post-SET	
	5	*SUVH5* (*SDG9*)	YDG, Pre-SET, SET, Post-SET	
	6	*SUVR1* (*SDG13*), *SUVR2* (*SDG18*), *SUVR4* (*SDG31*)	WIYLD, Pre-SET, SET, Post-SET	
	7	*SUVR5* (*SDG6*)	Pre-SET, SET, Post-SET	
VI. proteins with an interrupted SET domain; H3K36	NA	*ASHR1* (*SDG37*), *ASHR2* (*SDG39*), *ATXR1* (*SDG35*), *ATXR2* (*SDG36*), *ATXR4* (*SDG38*)	SET domain of ASHR1 interrupted by Zf-MYND domain	Restricts cell cycle progression
VII. RBCMT and other SET-related proteins; methylation of non-histone proteins	NA	*SDG40*, two anonymous proteins (corresponding to At2g18850 and At5g14260) and five uncharacterized proteins	SET domain	Carbon fixation

*OG-Number of orthology group; NA-not available.

Proteins with SET domain have actually been identified in chromatin-associated complexes that are formed during regulation of gene expression^25^. Through regulation of gene expression, SET domain proteins are also known to play a crucial role in diverse physiological processes in plants, including control of flowering time, leaf morphogenesis, floral organogenesis and seed development^26,27^. The genes encoding SET domain proteins that were the first to be characterized included the following: *CURLY LEAF* (*CLF*) and *MEDIA* (*MEA*), the latter also described as *FERTILIZATION INDEPENDENT SEED DEVELOPMENT 1* (*FISD1*) in *Arabidopsis thaliana*^24,28^. Characteristic features of plant SET domain proteins include chromatin binding and histone methylation that were first reported for proteins encoded by tobacco gene *NtSET1* and *Arabidopsis* gene *KRYPTONITE* (*KYP*)^29,30^.

The availability of complete genome sequences for many plant species allowed identification of families of SET domain genes in a number of species including *Arabidopsis thaliana*^21,23^, *Oryza sativa* (rice)^31^, *Zea mays* (maize)^32^, *Setaria italica* (foxtail millet)^33^, *Brassica rapa* (field mustard/turnip)^34^, *Vitis vinifera* (grapes)^35^ and *Gossypium raimondii* (cotton)^36^. The present study conducted for the first time in wheat, involved identification of 166 SET domain genes (SDGs), of which only 130 genes encoded proteins with complete SET domain (representing 117 unique genes excluding 13 duplicate genes). These genes were subjected to a systematic *in silic*o analysis, which included the study of gene structure, chromosomal distribution, gene duplication events, comparative genomics, promoter sequences and the presence of binding sites for miRNAs and genes for lncRNAs. The corresponding proteins were also subjected to a detailed study, which included the study of a variety of features including the following: (i) structure of proteins in terms of length and amino acid sequence; (ii) occurrence of functional domains and different classes of motifs; (iii) functional annotation; (iv) physicochemical properties, and (v) phylogenetic relationships. The study also included in silico analysis of expression of these genes in different tissues at different developmental stages under drought and heat stress using available expression database. Seven (7) representative SET genes were also used for qRT-PCR involving analysis of the expression of these SET domain genes under the following three contrasting conditions: (i) water stress using the two contrasting wheat genotypes, namely tolerant cv. C306 and sensitive cv. HD2967; (ii) heat stress using tolerant cv. HD2985 and sensitive cv. HD2329; and (iii) wheat-leaf rust infection, using a pair of NILs including the susceptible cv. HD2329 and its resistant NIL (carrying the gene *Lr28*). The study provides a strong base for further characterization and functional validation of SET domain genes in wheat.

## Results

During the present study, using reference wheat genome sequence, we identified and characterized 166 SDGs and described them as TaSDGs to specify that they belong to wheat. In the published literature, different numbering systems were used for different plant species (1–99 for Arabidopsis; 101–199 for maize, etc.). The 166 TaSDGs were labeled following numbering system used earlier for Arabidopsis^22,23^. Since only ~ 40 types of Arabidopsis genes were known and labeled as SDG1 to SDG40, additional numbers were used, wherever necessary, so that TaSDGs1 to TaSDG51 were available in the present study. Homoeologues were given the same numbers and distinguished using identity of homoeologous chromosomes (1A, 1B and 1D, etc.). Also, if TaSDGs having similarity to one Arabidopsis gene belonged to more than one homoeologous groups, these were distinguished by using alphabets a, b, c, etc. after the number (e.g. TaSDG34a, b, c, d).

### Identification of TaSDGs and their assignment to chromosomes

**Figure 1:**
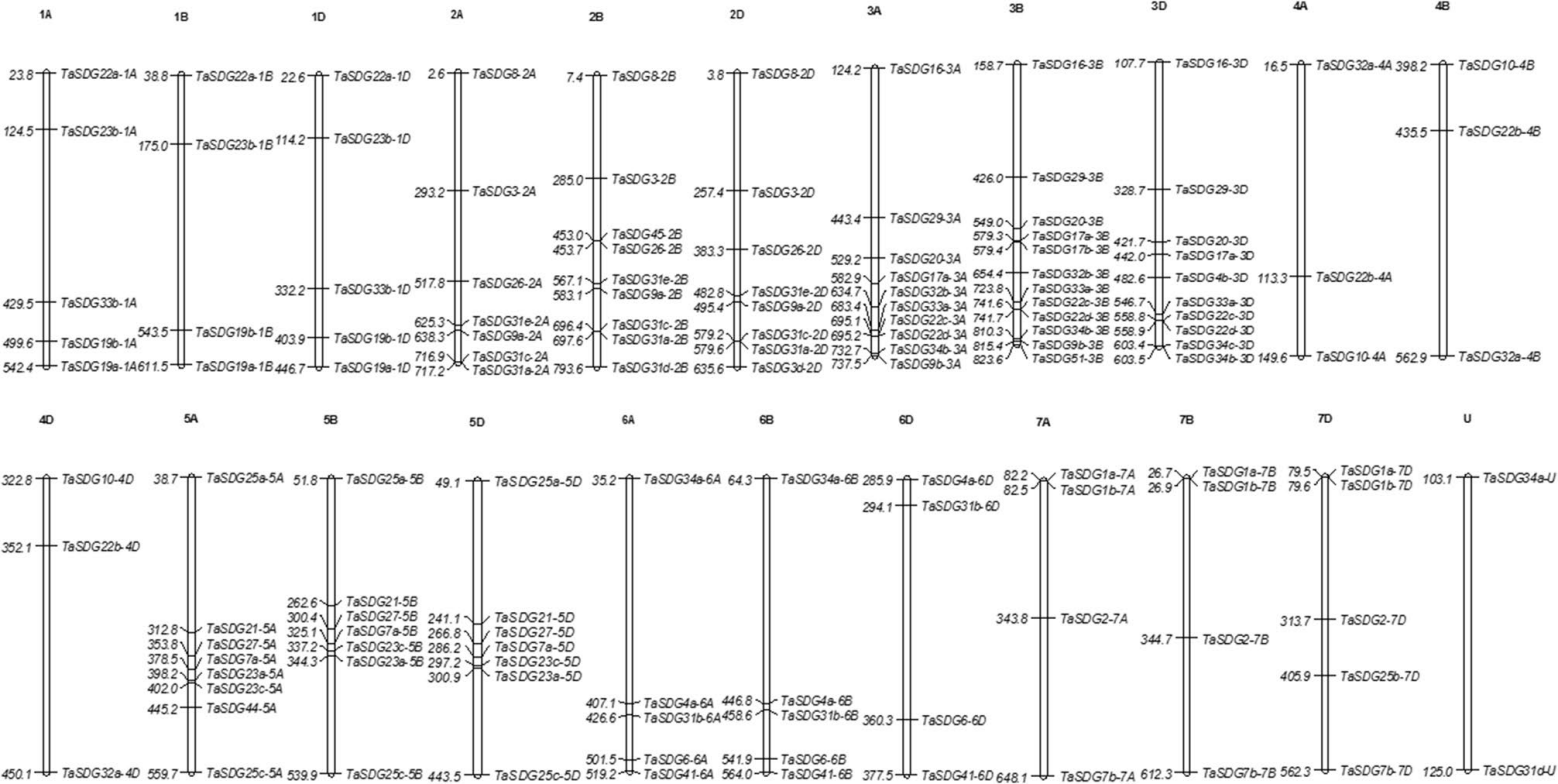
Chromosomal localization of TaSDGs on 21 chromosomes of wheat. The chromosome numbers are indicated on top of chromosomes. On each chromosome, the gene names are indicated on the right side and their physical positions are indicated on the left side. The TaSDGs were mostly located in the terminal regions and only a few TaSDGs were located in the sub-terminal or centric regions of different chromosomes. The figure was drawn using MapInspect software (https://www.plantbreeding.wur.nl/UK/ software_map-inspect.html).

The 166 TaSDGs identified as above, were placed in seven classes (class I–VII) on the basis of their similarity with SDGs in other diploid species (Supplementary Table S2). However, the proteins encoded by only 130 SDGs had full length SET domain; these 130 SDGs belonged to six of the seven (excluding class VI) classes and were distributed on all the 21 chromosomes [with three sub-genomes (A, B and D) and seven homoeologous groups (1–7)] (Fig. 1). Two genes, namely *TaSDG34a* and *TaSDG31d*, could not be assigned to any of the 21 chromosomes. Of the remaining 128 genes, the maximum number of genes were present in homoeologous group 3 (32), followed by group 2 (24), 5 (22), 6 and 1 (14 on each group), 7 (13) and group 4 (9). Among individual chromosomes, 3A carried the maximum of 12 genes, while 4A, 4B and 4D each carried a minimum of three genes. Almost equal number of genes were distributed on the three sub-genomes as follows: 44 genes on B sub-genome, 42 genes each on A and D sub-genomes (Fig. 1). Most genes were located in the terminal regions of chromosomes; only few genes were located in the sub-terminal or centric positions (Fig. 1). The above 128 SDGs could be placed in three sets, depending on their occurrence on all the three, or on only two or only one homoeologous groups: (i) 84 genes constituted 28 sets (each set with one gene on each of the three homoeologues); three TaSDGs belonging to each of these sets of homoeologues were homologous to one of the 20 SDGs of Arabidopsis, sometimes more than one set being homologous with the same Arabidopsis SDG. (ii) 12 genes comprised six sets of two homoeologues each distributed on two of the three sub-genomes of wheat (3 pairs on A/B, 2 pairs on A/D, and 1 pair on B/D), (iii) 6 genes had no homoeologues and were independently distributed on six individual chromosomes (5A, 2B, 3B, 3D, 4D and 7D) (Supplementary Table S2).

Of the 130 genes (each encoding protein with complete SET domain), 13 genes had duplicate copies; the duplications were either tandem or interspersed. The values of Ka (non-synonymous substitutions), Ks (synonymous substitutions) and Ka/Ks ratios for all the 13 duplicate (5 tandem and 8 interspersed) gene pairs is presented in Supplementary Table S3. The Ka/Ks ratio was < 1 for nine duplicate gene pairs, and was > 1 for three duplicate gene pairs (Ka/Ks ratio for the remaining one pair of duplicate genes could not be calculated, since Ka = Ks = zero). Estimates of timeline for divergence of duplicate genes were also calculated on the basis of Ka/Ks ratio, and were found to be in the range of 1.88–3.65 MYA for the origin of tandem duplications, and 1.65–6.65 MYA for interspersed duplications.

### Structure analysis of TaSDGs

Considerable variation was observed in the lengths of individual TaSDGs (867–22,640 bp), their corresponding cDNAs (819–7,516 bp) and CDSs (819–6,765 bp). Variation was also observed in the number of exons (1–24) and introns (0–23) in individual TaSDGs; 35 of the 73 TaSDGs in class V had no introns. Distribution of intron phases was as follows: phase 0 (58.41%), phase 2 (23.53%) and phase 1 (18.06%). Maximum number of genes (65) have all the three intron phases (0, 1, 2) followed by 14 genes having two phases (0 and 2); remaining 15 genes had one or two intron phases (Supplementary Figs. S1a-S1f.). Eighty five (85) TaSDGs each had only a single transcript while the remaining 45 genes had each 2–7 splice variants. The summary of the results of structure analysis of TaSDGs is presented in Table 2 and detailed information is available in Supplementary Table S4.

**Table 2:** A summary of the variation in the lengths of TaSDGs, cDNA and CDS belonging to six different classes in wheat.

Classes of genes	Range of gene lengths (bp)	Range of cDNA lengths (bp)	Range of CDS lengths (bp)	Range of number of exons/gene	Range of number of transcripts/gene
I	7,367–13,517	2,907–3,429	2,406–2,907	15–17	2–4
II	4,481–22,640	1,068–6,887	1,017–5,499	10–17	2–3
III	2,221–17,500	1,278–7,516	1,278–6,795	8–24	2–7
IV	1,483–3,946	819–1532	819–1,092	5–6	0
V	867–20,933	867–5,817	867–4,899	1–15	2–5
VII	2,306–9,223	1649–3,744	1,491–3,744	5–14	2

Promoter analysis allowed identification of elements for basal transcription (TATA box and CAAT box) as well as specific cis-regulatory response elements (light responsive, tissue specific, biotic and abiotic stress responsive) within 1 kb 5′ upstream sequence of each of the 130 TaSDGs. The details of these elements for basal transcription and specific response elements is provided in Supplementary Table S5. Eleven (11) response elements were identified which could be grouped as follows: (i) two response elements for biotic stress, namely GARE and TC rich repeat, and (ii) nine response elements for abiotic stress, namely, ARE, ABRE, P-box, CCATT, LTR, MBS, GARE, GC and TCA [one response element (GARE) was common between biotic and abiotic stresses]. These response elements were identified in 127 of 130 TaSDGs. However, tissue specific response elements were present in relatively fewer genes (33 of 130). Some response elements were present in multiple copies (Supplementary Table S6). Promoter sequences of only 47 of 130 genes had transcription factor binding sites (TFBS) related to nine families of transcription factors (ERF, C2H2, BBR-BPC, Dof, MIKC-MADS, MYB, GATA, NAC and Nin-like). Of these 47 genes, the promoters of 33 genes each had a single TFBS; promoters of the remaining 14 genes had 2–6 TFBS (Supplementary Table S7). TFBS for ERF was present in 25 genes followed by C2H2 (6 genes), BBR-BPC (5 genes), Dof (4 genes), MIKC-MADS/MYB (2 genes each). A solitary TFBS was present in each of the remaining genes and were meant for binding of TFs belonging to one of the following TF families i.e. GATA, NAC and Nin-like TF.

As many as 196 SSRs were detected in different genic regions (exons, introns, UTRs) of 96 of the 130 TaSDGs. The SSRs included mononucleotide to octanucleotide repeats. The number of SSRs per TaSDG varied from 1 to 10 (Supplementary Table S8). Trinucleotide repeats were most abundant (79 SSRs) followed by hexanucleotide repeats (47 SSRs), tetranucleotide repeats (24 SSRs), and others. A total of 42 TE were also identified in 25 of the 130 TaSDGs. These TEs were mainly DNA transposons (En/Spm) and retro-elements [LTR (Copia and Gypsy) and non-LTR (SINE)] (Supplementary Table S9).

Target sites for some miRNAs and gene sequences for some lncRNAs were also available in TaSDGs. Nearly 20% of TaSDGs (27/130) had target sites for 18 different miRNAs. The promoters of only two TaSDGs (*TaSDG22b-4D* and *TaSDG22c-3D*) each had target sites for two different miRNAs. The expression of TaSDGs with target sites for miRNAs were apparently inhibited through post-transcriptional cleavage except the following four miRNAs, which were found to inhibit expression of the target genes at the translational level: (i) tae-miR1120c-5p inhibiting genes *TaSDG1b-7D* and *TaSDG6-6A/B,* (ii) tae-miR1122b-3p inhibiting gene *TaSDG1b-7D*, (iii) tae-miR1137b-5p inhibiting gene *TaSDG17a-3A,* and (iv) tae-miR1130b-3p inhibiting the gene *TaSDG33b-1D* (Supplementary Table S10). Forty nine (49) of 130 TaSDGs also carried genes (or parts thereof) encoding as many as 122 lncRNAs, with a range of 1–10 lncRNAs within the same TaSDG, but majority of TaSDGs (24 of 49) each carried a gene for a single lncRNA. The length of gene sequences for lncRNAs ranged from 201–3,64,413 bp, the maximum size of lncRNA genes, sometimes exceeding the maximum lngth of TaSDG, so that the TaSDG carried only part of a gene for lncRNA (Supplementary Table S11).

### Structure analysis of TaSDG proteins

A summary of the details about lengths of proteins, their molecular weights and other important features are available in Table 3 (more details are available in Supplementary Table S12). Taken together, the number of positively charged amino acids was greater (26–1,339) relative to negatively charged amino acids (32–342). The TaSDG proteins also contained some important domains other than SET domain. These other domains included the following: AWS, WIYLD, Pre-SET, PHD, PWWP, FYRC, FYRN, Post-SET, YDG, Zf, CXC (Supplementary Table S13). It is on the basis of these domains that TaSDG proteins were grouped into six different classes (except class VI). TaSDGs within classes I-V were further classified into one (class IV) to seven (class V) orthology groups, as done in earlier studies^22,23^ (for details, see Supplementary Table S13).The distribution of the motifs in TaSDG proteins belonging to the six different classes (I-V and VII) is presented in Supplementary Table S14. The proteins within a class were also examined for common motifs, which ranged from 2 (class III) to 18 (class I). Among these motifs, some novel motifs were also identified; these novel motifs within a class ranged from one (class II) to 11 (class I) (for details of motif sequences, see Supplementary Table S14).

**Table 3:** A summary of the different chracateristics of proteins encoded by TaSDGs in wheat.

Class	Pr. length	Mol wt	PI	PR	NR	II	AI	GRAVY
	(Range)	(Range)	(Range)	(Range)	(Range)	(Range)	(Range)	(Range)
I	801–890	89.1–99.5	6.6–8.7	107–142	111–124	50–57	60.5–65.3	0.13
II	338–1,332	39.1–201.7	4.8–9.2	46–230	42–242	46–75	60.3–74.7	0.48
III	425–2,264	48.7–255.5	6.4–9.5	74–1,339	78–342	39–59	62.7–76.2	0.26
IV	272–363	30.80–40.1	8.7–9.0	41–52	36–45	49–66	74.3–82.0	0.21
V	288–1632	7.5–183.6	5.0–9.1	26–185	32–211	38–63	45.5–88.5	0.72
VII	496–1,247	55.2–140.6	4.6–9.0	48–167	60–213	46–56	78.4–97.8	0.44

Mol wt-Molecular weight; PI-Isoelectric point; PR-Positively charged amino acids; NR- Negitively charged amino acids; II- Instbility index; AI-Aliphatic index; GRAVY-Grand average of Hydropathy.

Gene ontology terms for predicted TaSDG proteins were classified into three well-known classes, namely biological process, cellular component and molecular function (Supplementary Fig. S2). Among the biological processes, most of the predicted TaSDG proteins were localized in the nucleus and were apparently involved in methylation of lysine residues of histone proteins (Supplementary Fig. S2); the proteins encoded by the following four genes belonging to class VII were located in chloroplast: *TaSDG41-6A/B/D* and *TaSDG44-5A*; these are involved in methylation of non-histone proteins such as Rubisco. The molecular functions of TaSDG proteins generally included the following: (i) zinc-ion binding, (ii) histone-lysine N-methyltransferase activity and (iii) protein binding (Supplementary Fig. S2).

### Phylogenetic analysis of TaSDG proteins

**Figure 2:**
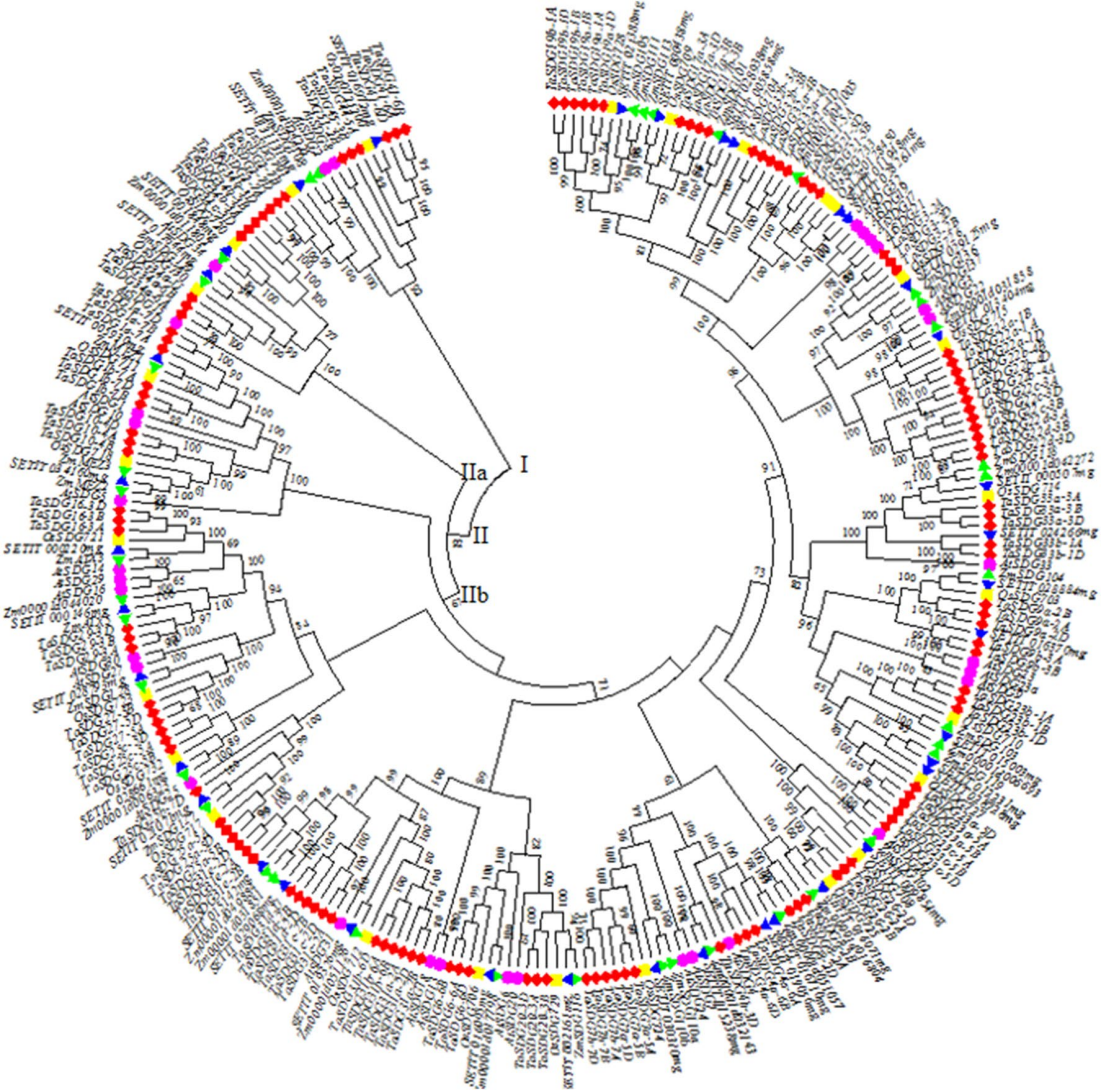
An un-rooted Neighbor-joining phylogenetic tree (created using MEGA version 6.0;^66^) showing relationship of TaSDG proteins with SDG proteins of *A. thaliana*, *O. sativa*, *Z. mays* and *S. italica*. The tree has two main clusters (cluster I and II). The cluster II is further divided into two sub-clusters IIa and IIb. Cluster IIb contains 13 sub-sub-clusters.

Phylogenetic tree prepared using aa sequences of SDG proteins of wheat, rice, maize, foxtail millet and Arabidopsis is presented in Fig. 2. The tree contains two major clusters, namely Cluster I and Cluster II. The Cluster I had all the SDG proteins belonging to class VII and SDG2 proteins (class III) for all the five species including wheat. The Cluster I also contained SDG8 protein (class II) of Arabidopsis. The Cluster II contained two sub-clusters IIa and IIb. The sub-cluster IIa contained SDG proteins of class IV belonging to all the five species including wheat. Similarly, sub-cluster IIb comprised 13 sub-sub-clusters, which contained SDG proteins belonging to different orthology groups of four classes, namely classes I to III and V for each of the five different species.

### In silico expression analysis of TaSDGs

**Figure 3:**
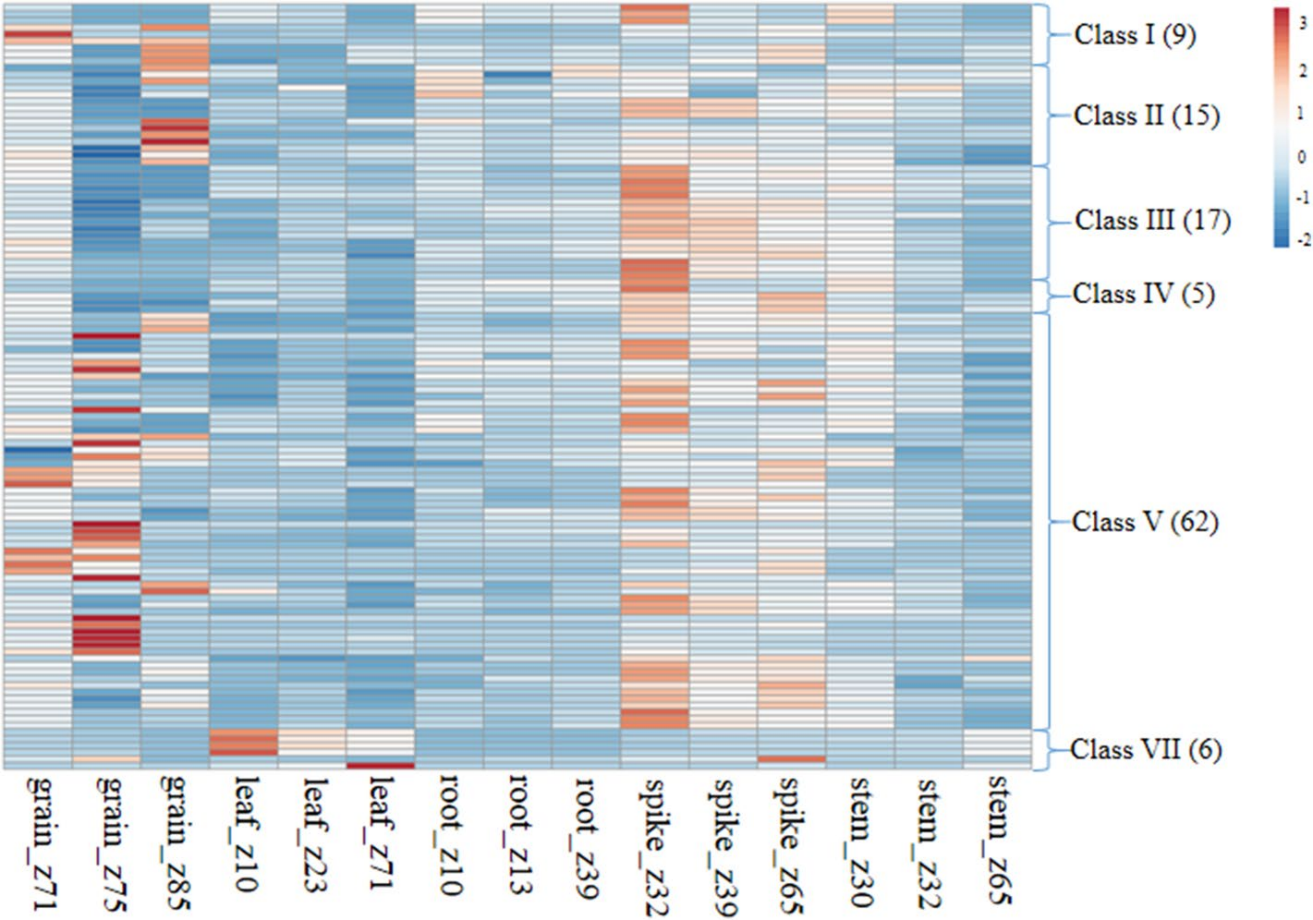
Heat map (generated using the online software tool ClustVis; https://biit.cs.ut.ee/clustvis/) showing in silico expression profile of 114 TaSDGs belonging to six classes at different developmental stages of five different tissues of wheat. The figures mentioned in parenthesis represent number of genes within a class. For further details of genes within each class see Supplementary Table S15.

The expression data for 114 of the 130 TaSDGs was available in the WheatExp database. The expression of these genes was examined in five different tissues (root, stem, leaf, spike and grain) sampled at different growth stages (according to Zadoks growth scale (Z00 to Z95) and under conditions of heat and drought. The summary data in terms of level of expression (up-regulation and down-regulation) is presented in Fig. 3; more details are available in Supplementary Table S15. Following expression results were particularly noteworthy: (i) very high expression (FPKM > 55) of *TaSDG4b-3D* in grain at Z85 stage and that of *TaSDG51-2B* in leaf at Z75 stage; (ii) tissue specific and developmental stage specific high expression (FPKM > 20) of the following genes: *TaSDG4b-3D* (grain_Z71/85, leaf_Z71 and root_Z10), *TaSDG31e-2B* (spike_Z32/39/65), *TaSDG31c-2D* (spike_Z32), *TaSDG41-6A/B/D* (leaf_Z10), *TaSDG44-5A* (leaf_Z10) and *TaSDG51-2B* (leaf_Z71).

**Figure 4:**
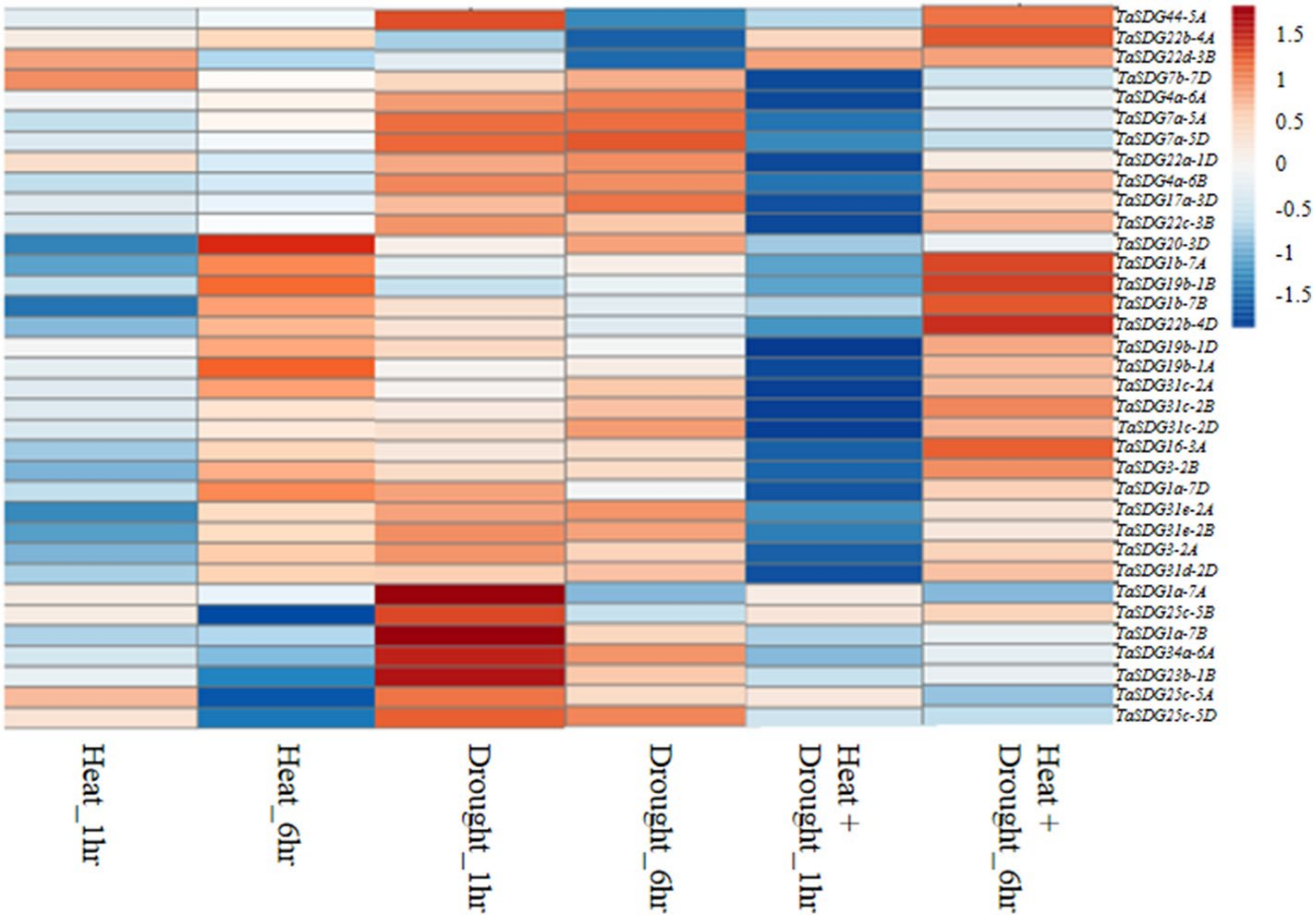
Heat map (generated using the online software tool ClustVis; https://biit.cs.ut.ee/clustvis/) showing in silico expression profile of TaSDGs (fold change ± 2) under heat, drought and combined stress of heat and drought.

As many as 36 of 114 genes responded to heat and drought stress at the seedling stage and their expression pattern changed by ± twofold under heat/drought (Fig. 4, Supplementary Table S16). Many more genes were down-regulated (30 genes; range of fold change:-2.0 to -5.39) relative to the number of genes that were up-regulated (6 genes; range of fold change: 2 to 4.58). Under heat stress, the number of genes showing differential expression ranged from 6 genes (one up-regulated and five down-regulated) at 1 h to 13 genes (four up-regulated and nine down-regulated) at 6 h; one gene (*TaSDG1a-7B*) was common under both the treatments. Under drought, the number of differentially expressed genes ranged from 2 (both genes showed up-regulation) at 1 h to 3 (all three showed down-regulation) at 6 h. The results were opposite under the combined heat and drought stress, so that the number of differentially expressed genes included one up-regulated and 23 down-regulated genes at 1 h; four genes up-regulated and five genes down-regulated at 6 h (four genes were common under 1 h and 6 h; one up-regulated [*TaSDG22b-4A*] and three down-regulated [*TaSDG1a-7B, TaSDG4b-3D and TaSDG25c-5D* ]). The details of these differentially expressed genes and the levels of expression are shown as heat maps in Fig. 4.

### qRT-PCR analysis in response to heat, drought and leaf rust

For validating the results of in silico expression analysis, seven representative genes were selected and their expression was examined in three pairs of contrasting genotypes, one pair for each stress. However, qRT-PCR data was available for all the seven genes for heat stress, for six genes under water stress, and for only two genes for leaf rust infection. The results of differential expression obtained using qRT-PCR are summarized in Table 4.

**Table 4:** A summary of the results of qRT-PCR analysis for seven TaSDGs in two contrasting wheat cultivars each under water stress and heat stress and in a pair of NILs for leaf rust gene *Lr28*.

Description of TaSDG	Water stress 1 h		Water stress 6 h		Heat stress		Leaf rust (96hai)	
	HD2967 (S)	C306 (T)	HD2967 (S)	C306 (T)	HD2329 (S)	HD2985 (T)	HD2329 (S)	HD2329 + *Lr28* (R)
*TaSDG1a−7A* (class I; H3K27)	NS	NS	↑(2.14)	NS	↓ (− 3.87)	↓ (− 3.16)	↓ (− 31.42)	↑ (8.68)
*TaSDG16-3A* (class III; H3K4)	NS	↓ (− 2.33)	NS	NS	NS	NS	–	–
*TaSDG22a-1D* (class V; H3K9)	NS	↓ (− 5.06)	NS	↓ (− 3.82)	NS	NS	–	–
*TaSDG20-3D* (class V; H3K9)	NS	NS	↓(− 2.46)	NS	↑ (2.85)	↑ (2.24)	↓ (− 6.46)	↑ (2.63)
*TaSDG25c-5D* (class V; H3K9)	–	–	–	–	↓ (− 3.78)	↓ (− 2.4)	–	–
*TaSDG44-5A* (class VII; methylation of non-histone proteins)	NS	NS	NS	NS	↓ (− 3.62)	NS	–	–
*TaSDG51-2B* (class VII; methylation of non-histone proteins)	NS	↑ (4.17)	NS	↓ (− 2.13)	↓(− 2.64)	NS	–	–

T: tolerant; S: sensitive/susceptible; R: resistant; 96hai: 96 h after inoculation; ↑: 2.14 to 8.68 fold upregulation; ↓: 2.13 to 31.42 fold downregulation; NS: non-significant expression; -: despite repeated attempts qRT-PCR was not successful.

## Discussion

During the present study, we identified and characterized 166 TaSDGs using reference wheat genome sequence. However, complete sequence for SET domain was available in only 130 of these genes. The 166 TaSDGs were classified into seven widely known classes (I-VII) following the nomenclature of SDGs initially used in Arabidopsis. The proteins encoded by genes belonging to classes VI and VII in Arabidopsis contain only truncated or incomplete SET domain; the genes of class VII have not been given the SDG nomenclature in Arabidopsis (except SDG40), but only their IDs are available. The 130 TaSDGs, each encoding protein with complete SET domain, belonged to six (I to V and VII) of the seven well-characterized classes of SDGs^23^. The SDGs belonging to class VI encoded proteins with incomplete SET domain; hence were not analyzed further during the present study. However, six genes belonging to class VII encoded proteins, which had complete SET domains (unlike Arabidopsis) and therefore were included in detailed study; TaSDG nomenclature was given to these six genes belonging to class VII also (Supplementary Table S2).

It may be recalled that during the present study, the number of TaSDGs with full length SET domain in hexaploid wheat was 130, which is more than four times the number in each of the following diploid species: 27 in rice, 39 in maize, 37 in foxtail millet and 31 in Arabidopsis (Supplementary Table S17). Thus the number in hexaploid wheat exceeds even the expected three times the number in diploid species maize with the highest number of SDGs among the four diploid species examined. This may be attributed to availability of some duplicate genes in wheat, which might have originated during the course of two-step evolution of wheat^37,38^, although interspersed duplications of SDGs have also been reported in the above diploid species^31,32,36^. Particularly, in maize, one would expect duplications, since it has been shown to be a tetraploid on the basis of data on reference whole genome sequence of maize^39^. Other diploid species have also been shown to be palaeo-polyploids, so that duplications are common even in diploid species. Origin of duplicate gene is also a widely discussed subject and does not deserve any detailed discussion. Most of these duplicate genes in the present study belong to class V, a feature that has also been observed in maize and Arabidopsis^22^. The evolutionary time-line suggested that the tandem duplications (range: 1.88–3.65 MYA) are of more recent origin relative to the interspersed duplications (range: 1.65–6.65 MYA). However, Ka/Ks ratio of most of the tandem duplicate gene pairs was > 1 indicating positive selection on these genes contributing to molecular evolution^40^. The interspersed duplications, on the other hand, had Ka/Ks ratio < 1, indicating that these gene pairs are under purifying selection. The duplication events are known to give rise to new genes and create functional novelty in any organism^41^.

In hexaploid wheat, we expect three homoeologues for each gene, although this is not true for all genes. In the present study also, there were 18 TaSDGs, which did not have all the three homoeologues. However, these genes with missing homoeologues are available in the diploid (AA and DD) and tetraploid (AABB) wheat progenitors, suggesting that the missing genes might have been eliminated during the course of evolution of the hexaploid wheat. Otherwise also, gene loss has been reported as a common phenomenon during the course of evolution of hexaploid wheat from its diploid progenitors^42^.

Another interesting feature of the present study is the absence of some wheat homologues of Arabidopsis SDGs^19^ including the following: (i) *MEA* gene (class I-OG1); (ii) *SDG24* (class II-OG1), (iii) *SDG30* (class III-OG1), (iv) *SDG14* (class III-OG2) and (v) *SDG15* (class IV-OG1) (for details see Supplementary Table S2). Similar results were also reported in some other monocots including rice, maize and foxtail millet^31–33^. It might be possible that the functional diversification of homologs of SET domain genes occurred after the divergence of monocots and dicots ~ 200 MYA^32^. Some of the missing homologs in the monocots (wheat, rice, maize and foxtail millet) were perhaps lost after their divergence from other dicots like Arabidopsis. Future studies may provide answer to this problem.

Structural analysis of TaSDGs also revealed some interesting features including the following: (i) Enormous variation in the length of individual TaSDGs (867–22,640 bp), which is also reflected in the lengths of corresponding proteins (272aa to 2264aa). This is not surprising, since the length (22.64 kb) of the longest wheat gene *TaSDG7a-5B* is still smaller than the longest SDG reported in maize (44.5 kb)^32^ and that the length of SDG proteins also varied in several diploid species [foxtail millet (301–2267aa), rice (298-2257aa), maize (173-1815aa) and Arabidopsis (203–2,351 aa)]^23,31–33^. The variation in the length of SDGs is mainly due to the number of introns and their relative lengths, and not due to number of exons, suggesting the occurrence of same coding potential in different SDGs in wheat and other species; the codons also appear to be conserved, as apparent from high frequency of intron phase 0 (58.4%)^43^. The variation in translation products, however seems to result from variation in the number of splice variants (2–6) and alternate splicing^44–46^. (ii) Presence of YDG domain in TaSDGs belonging to class V-OGs 1, 2, 3, and 5; (iii) Absence of introns except for the five genes (belonging to class V), namely *TaSDG33a-3A/B/D* and *TaSDG33b-1A/D* (each containing 14–15 introns; for details see Supplementary Fig. S1e), which were homologs of Arabidopsis gene *SDG33* with four introns. The observed absence of introns in most of the class V SDGs in wheat, maize and Arabidopsis might be due to an ancient retro-transposition-like event that occurred before the divergence of monocots like wheat and maize and dicots like Arabidopsis^22^; (iv) Presence of complete SET domain in six TaSDG proteins derived from class VII genes (*TaSDG41-6A/B/D*, *TaSDG44-5A*, *TaSDG45-3B* and *TaSDG51-2B*); the proteins derived from class VII SET domain genes generally carry truncated SET domain in Arabidopis and other species. Surprisingly, in the diploid and tetraploid progenitors of wheat also, five of the six TaSDGs (except *TaSDG45-3B*, which had complete SET domain) lacked complete SET domain. Therefore, it appears that the evolution of the above complete SET domain containing TaSDGs occurred after the evolution of the hexaploid wheat. Some of these SDGs are believed to be involved in the methylation of non-histone proteins. For instance, the genes *TaSDG41-6A/B/D* and *TaSDG44-5A* encode Rubisco small sub-unit methyltransferases (RSSMT) and Rubisco large sub-unit methyltransferases (RLSMT), respectively. The RLSMT is known to methylate lysine 14 in the large subunit of Rubisco protein while the RSSMT is known to methylate the methionine in the small subunit of Rubisco protein^47^.

Other interesting features of TaSDGs recorded in the present study include occurrence of SSRs, transposon elements (TE), target sites for some miRNAs and genes (complete or part thereof) for lncRNAs; these will be briefly discussed one-by-one. First, the presence of SSRs can lead to phenotypic variation, since SSRs affect several processes including transcription, translation, mRNA splicing, export to cytoplasm, and loss of function^48^; polymorphism in SSRs may also be used for molecular breeding, once we know the association of specific SSRs to the target traits. Second, the TE including En/Spm, Copia, Gypsy and SINE, which occur in 20% TaSDGs, may help in bringing about epigenetic changes during heat stress, as shown in Arabidopsis mutant for *suvh2/SDG18* gene (deficient in H3K9 methyltransferase activity)^49^. Third, a number of TaSDGs have been shown to be the targets of miRNAs. From among 18 miRNAs for which target sites were available in TaSDGs during the present study, miR1135 and miR5049-3p are known to occur in Brachypodium and miR5049-3p occurs in *Saccharum*. The target sites of different miRNAs obviously differed. For instance, different miRNA differ for regions of the target genes (3′UTR, 5′UTR, promoter) with which they interact; miR1137a shows interaction with UTR of *TaSDG22b-4D* and tae-miR1127a shows interaction with promoter of *TaSDG22c-3D*; this information is important because binding of miRNAs to 5′UTR is known to have silencing effects^50,51^, whereas miRNA interaction with promoter region is known to induce transcription^52^. Interaction of miRNAs with 3′UTR of their target mRNAs (resulting in translational repression and mRNA deadenylation and decapping) has also been reported, in several earlier studies^53,54^. However, functions of some of the miRNAs, namely miR1120c-5p, tae-miR1130b-3p, tae-miR1120b-3p and tae-miR5049-3p having TaSDGs as their targets are known to regulate transcription leading to their effect on flower development and pollen recognition^55^; this information, along with other information about miRNAs, may be utilized in designing strategies for using miRNA for wheat improvement. Future experiments may also be designed to understand the mechanism of action of miRNAs. Fourth, the 122 lncRNAs, for which genes were available in 49 TaSDGs provide useful information for further detailed study, since a number of lncRNAs are known to mediate epigenetic changes by recruiting chromatin-remodeling complex to specific genomic loci. For instance, COOLAIR and COLDAIR lncRNAs are necessary for recruiting PHD-PRC2 complex to enable histone modifications of *FLC* (a key regulator of flowering time) in Arabidopsis, which acts as a repressor to inhibit flowering under cold temperature^56^.

In addition to the widely known structure of SET domain proteins including the presence of SET domain and their function as HMTases, these proteins may perform other important functions including those due to a number of other domains (detected during the present study) including PHD and PWWP domains^57,58^. These other functions can be resolved only through a study of their high resolution structure, which needs availability of these genes in crystalline form. Unfortunately, all TaSDG proteins are unstable and hydrophilic in nature (except TaSDG25b-7D and TaSDG31b-U), as evident from the values of their aliphatic indices (45.5– 97.8)^59,60^ and GRAVY values (− 0.16 to − 0.811). A detailed study of all TaSDG proteins is therefore necessary to make full use of these genes in wheat improvement programmes.

The results of phylogeny also provide some interesting feature, although evolutionary patterns appear to be largely conserved. It may be seen from the results that Cluster I included SDG2 proteins of class III (for all species examined), including the three SDG2 proteins of wheat (TaSDG2-7A/7B/7D); these were however grouped with proteins from class VII TaSDGs, which may be attributed to high similarity of TaSDG2-7A/7B/7D with class VII SDG proteins (including presence of no other domain except SET domain). Since contrary to expectation, class VII TaSDGs carried complete SET domain, we were expecting that the clustering pattern of TaSDG proteins may also show some other important differences from those in Arabidopsis, rice, maize and foxtail millet. However, no such difference was observed in the clustering pattern of SDG proteins in the present study and earlier studies in a number of dicots and monocots (including Arabidopsis, foxtail millet, maize, rice, mustard/turnip and diploid wild cotton)^21,31–34,36^. Further investigations may help to find out the reason for the occurrence of complete SET domain in class VII wheat SDG proteins, and that of incomplete SET domain in class VII SDG proteins of other species.

Expression of TaSDGs in time (development stages) and space (different tissues) also provided some interesting results, particularly when expression results were examined along with information about the occurrence of some regulatory cis-elements in TaSDGs. This was necessary since SDGs are known to play a major role in plant development and also in response to different biotic and abiotic stresses including hormonal treatments^13,31,33,61^. In the present study, cis-elements were found to be present in almost all wheat TaSDGs with some exceptions (*TaSDG31c-2B*, *TaSDG6-6B* and *TaSDG29-3A*). These cis-elements include those, which are the binding sites for some important transcription factors and thus also respond to biotic and abiotic stresses (GARE and TC rich elements for biotic stresses; ARE, ABRE, P-box, CCATT, LTR, MBS, GARE, GC and TCA for abiotic stresses). Perhaps these regulatory cis-elements respond to different developmental cues and stresses through expression of these TaSDGs in the form of HTMases, which bring about histone methylation as also mentioned in the Introduction^62^. The expression of these TaSDGs in response to biotic and abiotic stresses is mediated through activation of a number of transcription factors (mentioned in “Results”), for which binding sites occur in these TaSDGs. It may also be recalled that under stress, as many as 30 TaSDGs were down-regulated, but only six were up-regulated in different plant organs such spike, grain, leaf, stem and roots. The six up-regulating genes (*TaSDG19b-1A*,-*1B*,-*1D*, *TaSDG23b-1B*, *TaSDG22b-4A* and *TaSDG22b-4D*) belong to class V and are known to be involved in methylation of H3K9. This epigenetic mark is likely to repress the expression of genes that positively respond to heat (*TaSDG19b-1A*), drought (*TaSDG23b-1BI*) and heat + drought (*TaSDG19b-1B*,-*1D*, *TaSDG22b-4A* and *TaSDG22b-4D*).

The results of in silico expression analysis could be validated through qRT-PCR at least for some genes (Supplementary Table S16, Table 4). Five of the seven genes used for qRT-PCR are involved in methylation of H3K4, 9 and 27 and the remaining two genes (*TaSDG44-5A* and *TaSDG51-2B*) are involved in methylation of non-histone protein (Table 4). Following are some important conclusions involving differential expression of TaSDGs, which may be involved in methylation of specific lysine residues of H3 protein and may respond to water stress, heat stress and leaf rust: (i) Under water stress, *TaSDG1a-7A* is up-regulated in sensitive cultivar HD2967; (ii) Under heat stress, *TaSDG20-3D* is up-regulated in both the sensitive (HD2329) and tolerant (HD2985) cultivars; (iii) During leaf rust infection, two genes (*TaSDG1a-7A* and *TaSDG20-3D*) showed significant up-regulation in resistant NIL (HD2329 + *Lr2*8) 96 h after inoculation with leaf rust. The genes *TaSDG1a-7A* (class I) and *TaSDG20-3D* (class V) respond to all the three stresses including water stress, heat stress and leaf rust resistance due to *Lr28*. Since it is known that SDGs belonging to class I and V are involved in methylation of H3K9 and H3K27^23^, and that both these epigenetic histone marks suppress gene expression, it appears that the expression of these two genes is induced by these abiotic and biotic stresses which may indirectly be involved in downregulation of genes providing tolerance to these stresses. Therefore, the genes *TaSDG1a-7A* and *TaSDG20-3D* with their cis-regulatory elements may prove useful for improvement of stress tolerance in wheat. This received support from the results of our other studies, where a set of genes carrying domains of bHLH TF, auxin response factor, F-box, etc. were associated with high affinity differential binding sites of H3K27me3 (a repressor mark) in resistant NIL (HD2329 + *Lr28*). This binding perhaps acts as negative regulators of leaf rust resistance^63^.

## Materials and methods

### Identification of SET domain genes in wheat and their homologs in other plant species

Following different approaches were used to identify putative SET domain genes (SDGs) from wheat: (*i*) BLASTP search against wheat proteome (https://plants.ensembl.org/Triticum_aestivum/Tools/Blast?db=core) containing amino acid sequences of wheat proteins; these were downloaded from Pfam database using Pfam ID (PF00856) of SET domain; (ii) tBLASTx search against the wheat genome (https://plants.ensembl.org/Triticum_aestivum/ Tools/Blast?db = core) using known CDS sequences of SDGs of rice, maize, Arabidopsis and *Setaria* (containing nucleotide sequences corresponding to SET domain); (iii) Keyword search using ‘SET domain’, conducted in EnsemblPlants and, (iv) HMMER tool (available at EnsemblPlants) used to retrieve additional genes. The hits retrieved from the above methods were examined for the presence of SET domain using conserved domain database (CDD) batch search tool at NCBI (https://www.ncbi.nlm.nih.gov/Structure/bwrpsb/bwrpsb.cgi).

Wheat SDGs (TaSDGs) identified as above were checked for their homologs in rice, maize, Arabidopsis and *Setaria*. The sequences having complete SET domains were then used as query in TblastN against EnsemblPlants to retrieve all the information (gene, transcript, splice variants, cDNA, CDS and protein) related to corresponding SDGs in wheat. Homoeologous relationships between SDGs of wheat were established on the basis of their chromosome assignment and percentage of protein sequence identity (> 90%). TaSDGs were named following the classification of SDGs in Arabidopsis^23^.

### Physical map of TaSDGs and identification of duplicate genes

Information regarding chromosome location and the coordinates for individual SDG of wheat was obtained from EnsemblPlants database (https://mar2016plants.ensembl.org/Triticum_aestivum/Info/Index). Physical map of TaSDGs was prepared using MapInspect software (https://www.plantbreeding.wur.nl/UK/ software_map-inspect.html). In order to identify gene duplications, CDS sequences of TaSDGs were blasted against each other and the genes having > 90% identity were accepted as duplications^64^. If two or more than two genes were found to be located on the same chromosome adjacent to each other, these genes were treated as tandem duplications^65^.

### Ka/Ks analysis

Synonymous substitutions (Ks) and non-synonymous substitutions (Ka) were calculated for duplicated gene pairs using MEGA 6.0^66^ software. Ka/Ks ratio of < 1 suggested purifying selection, Ka/Ks ratio of > 1 suggested positive selection and Ka/Ks ratio was used to infer neutral selection. The time of duplication and divergence in terms of million years ago (Mya) for each duplicate gene pair was also calculated using a synonymous mutation rate of λ substitutions per synonymous site per year as (T) = Ks/2λ × 10^−6^ (λ = 6.1 × 10^–9^).

### Analysis of TaSDG nucleotide sequences

In order to analyse the structure of TaSDGs, the full length CDSs of TaSDGs were compared with their corresponding genomic sequences; Gene Structure Display Server (GSDS) v2.0 (https://gsds.cbi.pku.edu.cn/) was used for this purpose^67^. Identification of intron phases (0, 1, 2) was done using criteria that were used in our earlier studies^68,69^. The presence of cis-regulatory response elements was checked in one kb genomic region 5′ upstream of the translation start site (ATG) (i.e. promoter region) of each gene using PlantCARE database (https://bioinformatics.psb.ugent.be/webtools/plantcare/html/)^70^ following the criteria used by us in our earlier studies^71,72^. Transcription factor binding sites (TFBS) in the promoter region of each gene were predicted using PlantRegMap (https://planttfdb.cbi.pku. edu.cn/prediction.php)^73^. BatchPrimer3v1.0 (https://probes.pw.usda.gov/ batchprimer3/) was used to identify simple sequence repeats (SSRs) and transposable elements (TEs) within the gene sequences. The miRNAs and their targets in TaSDGs and their promoters were predicted employing web-based psRNATarget server (https://plantgrn.noble.org/psRNATarget/)^74^ using default parameters; the range of e-value was 0–2. The TaSDGs were also analysed for the presence of sites for lncRNAs using IWGSC database (https://urgi.versailles.inra.fr/jbrowseiwgsc/gmod_jbrowse/?data=myData%2FIWGSC_RefSeq_v1.0).

### Analysis of TaSDG protein sequences

The physicochemical properties of TaSDG proteins were studied using ExPASy ProtParam tool (https://web.expasy.org/protparam/). Major domains in the predicted protein sequences were identified through PROSITE (https://prosite.expasy.org/) and conserved domain (CD)-search program of conserved domain database (CDD) at NCBI (https://www.ncbi.nlm.nih.gov/Structure/bwrpsb/bwrpsb.cgi). Common motifs in proteins of individual class (I-V and VII) were identified using online motif finding tool MEME (Multiple Expectation Maximization for Motif Elicitation, v3.5.454) (https://meme-suite.org/tools/meme)^75^, using the option of 0 or 1 for a specific motif, and setting the upper limit of the number of motifs as 20, with an optimum length of each motif set at 6–50 amino acids. All identified motifs were annotated using InterProScan database (https://www.ebi.ac.uk/Tools/pfa/iprscan/). The TaSDGs were functionally annotated using BioMart available at EnsemblPlants. Gene ontology (GO) terms were classified into the following three well known classes: cellular component, molecular function and biological process.

### Phylogenetic analysis of SET domain containing proteins in wheat

Based on amino acid sequences of SET domain containing proteins, an un-rooted phylogenetic tree was constructed using MEGA version 6.0^66^ employing Neighbor-joining method of distance matrix, with a bootstrap involving 1,000 iterations using p-distance substitution model. The phylogenetic tree involved SET domain containing proteins from the following plant systems: wheat (130), rice (27), maize (38), Arabidopsis (33) and foxtail millet (37). All these protein sequences were aligned by multiple sequence alignment (MSA) tool available in MEGA version 6.0; the aligned files were used to generate a phylogenetic tree.

### In silico expression analysis of TaSDGs

The in silico expression analysis of TaSDGs in five different tissues (grain, leaf, root, spike and stem) each sampled at three developmental stages and during major abiotic stresses (heat, drought and heat + drought [1 h and 6 h stress]) was carried out using publicly available transcriptome data at wheat expression database (https://wheat.pw.usda.gov/WheatExp/). The online software tool ClustVis (https://biit.cs.ut.ee/clustvis/) was used to generate the heat maps. For this purpose, normalized gene expression values which are expressed as the number of fragments per kilobase of exon per million fragments mapped (FPKM), were transformed using log_2_.

### qRT-PCR for validation of in silico expression of TaSDGs

Expression of seven representative TaSDGs using qRT-PCR was also examined at the seedling stage in pairs of contrasting genotypes in response to abiotic stresses (water and heat) and biotic stress (leaf rust). The genes were selected on the basis of results of in silico expression analysis during water and heat stresses. However, although in silico expression data was not available for leaf rust, qRT-PCR was also conducted for leaf rust, to find out the role of TaSDGs during leaf rust infection. The seven genes included the following: *TaSDG1a-7A* (class I), *TaSDG16-3A* (class III), *TaSDG22a-1D*, *TaSDG20-3D*, and *TaSDG25c-5D* (class V) and *TaSDG44-5A* and *TaSDG51-2B* (class VII). Primers for these selected genes were designed using Primer3 software (Supplementary Table S1). The analysis was conducted using the material and methods that were used in our earlier study^72^. Briefly, following three pairs of contrasting genotypes were utilized and were subjected to three different stresses as follows: (i) For water stress, samples were taken from seedlings of a pair of genotypes (tolerant cv. C306 and sensitive cv. HD2967) that were subjected to 1 h and 6 h of water stress. (ii) For heat stress, samples were taken from tolerant cv. HD2985 and sensitive cv. HD2329 that were subjected to 2 h of heat stress. (iii) For leaf rust, samples were collected at 0 h before inoculation (0hbi) and 96 h after inoculation (96hai) from a pair of NILs including susceptible cultivar HD2329 and its resistant NIL HD2329 + *Lr28* that were inoculated with virulent race of the pathogen (77–5). The material for three qRT-PCR experiments were collected as described in an earlier study^72^. Water stress was given by transferring the seedlings to modified Hoagland’s solution containing 20% PEG 8,000. Similarly, heat stress given by exposing 7 days old normal wheat seedlings to 42 °C for 2 h; The heat stress was given in a sinusoidal mode by increasing 1 °C temperature per 10 min till the temperature reached 42 °C, which was maintained for 2 h; seedlings at 22 °C were used as control. For leaf rust, the material was collected as described in an earlier study^72^. For each treatment in each experiment, two replications were used.

## Supplementary information

Supplementary information 1

Supplementary information 2

## Data Availability

All data generated or analysed during this study are included in this published article (and its Supplementary Information files).
