# Supplementary material for: Identification and characterization of SET domain family genes in bread wheat (Triticum aestivum L.)
Source: Sci Rep. 2020 Sep 3;10:14624. doi: 10.1038/s41598-020-71526-5 (PMC7471321; doi:10.1038/s41598-020-71526-5)
Supplement: Supplementary file 1 — Supplementary information 1 [file 41598_2020_71526_MOESM1_ESM.pptx]

## Slide 1
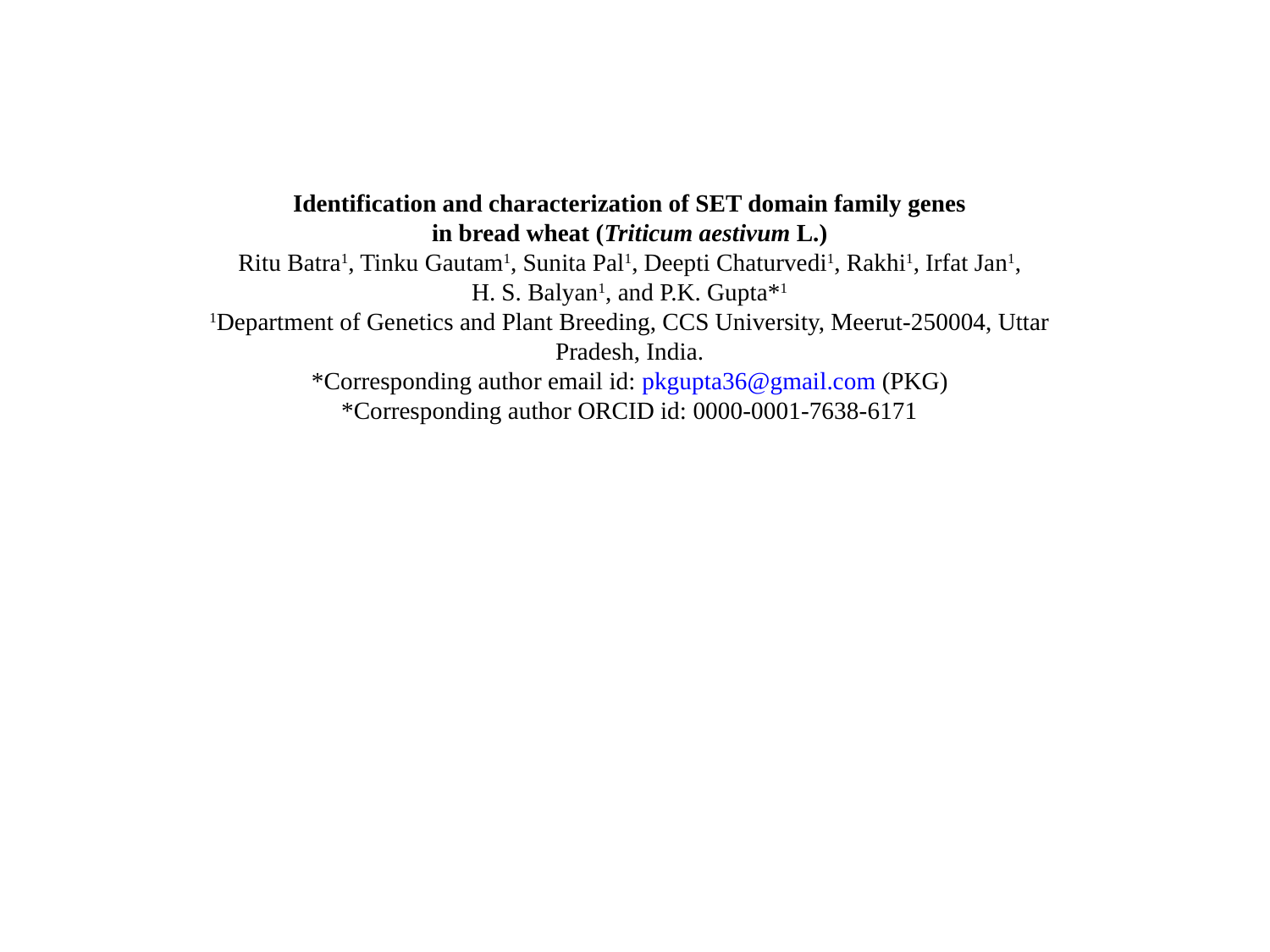

Identification and characterization of SET domain family genes
in bread wheat (Triticum aestivum L.)
Ritu Batra1, Tinku Gautam1, Sunita Pal1, Deepti Chaturvedi1, Rakhi1, Irfat Jan1,
H. S. Balyan1, and P.K. Gupta*1
1Department of Genetics and Plant Breeding, CCS University, Meerut-250004, Uttar Pradesh, India.
*Corresponding author email id: pkgupta36@gmail.com (PKG)
*Corresponding author ORCID id: 0000-0001-7638-6171

## Slide 2
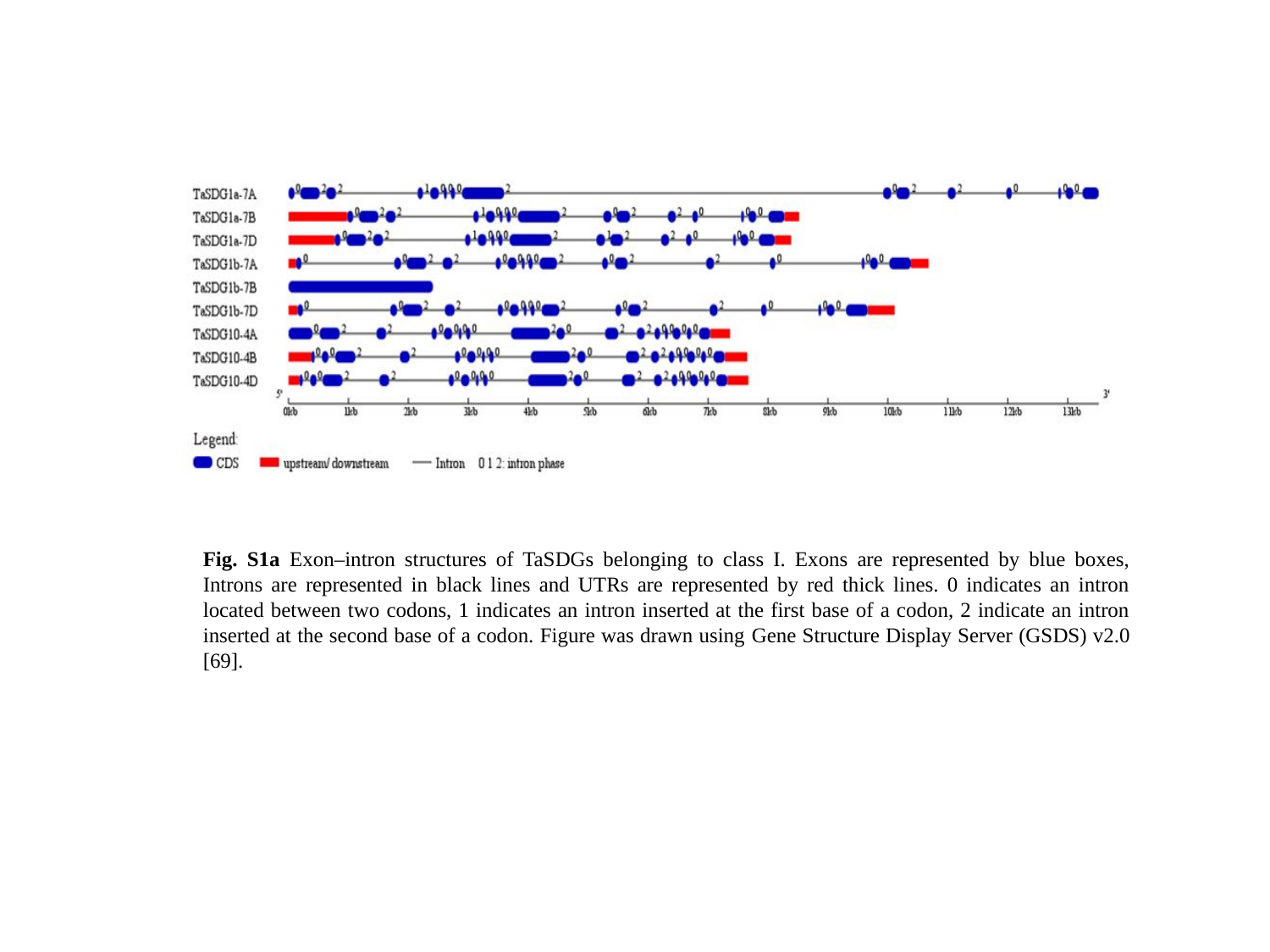

Fig. S1a Exon–intron structures of TaSDGs belonging to class I. Exons are represented by blue boxes, Introns are represented in black lines and UTRs are represented by red thick lines. 0 indicates an intron located between two codons, 1 indicates an intron inserted at the first base of a codon, 2 indicate an intron inserted at the second base of a codon. Figure was drawn using Gene Structure Display Server (GSDS) v2.0 [69].

## Slide 3
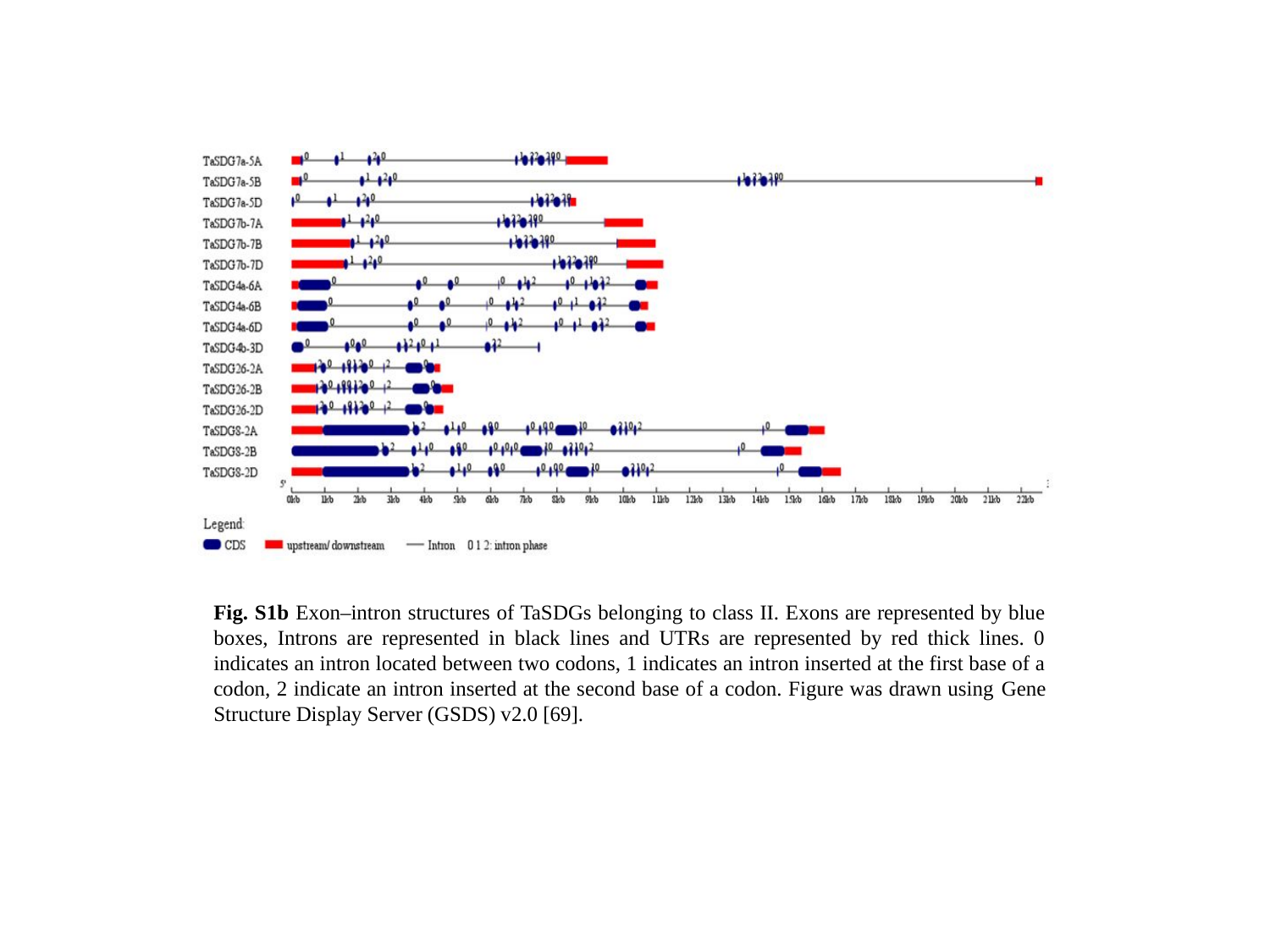

Fig. S1b Exon–intron structures of TaSDGs belonging to class II. Exons are represented by blue boxes, Introns are represented in black lines and UTRs are represented by red thick lines. 0 indicates an intron located between two codons, 1 indicates an intron inserted at the first base of a codon, 2 indicate an intron inserted at the second base of a codon. Figure was drawn using Gene Structure Display Server (GSDS) v2.0 [69].

## Slide 4
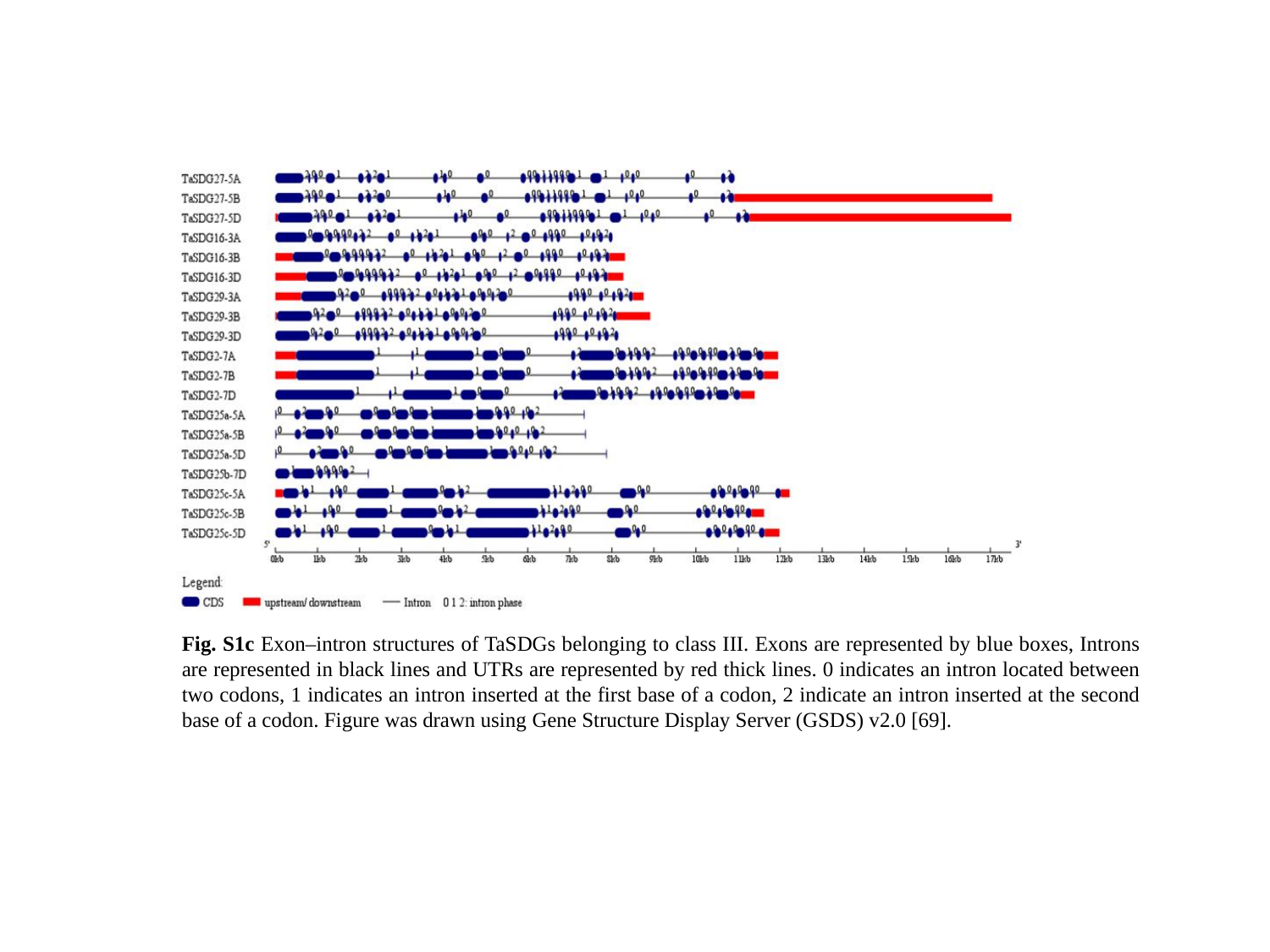

Fig. S1c Exon–intron structures of TaSDGs belonging to class III. Exons are represented by blue boxes, Introns are represented in black lines and UTRs are represented by red thick lines. 0 indicates an intron located between two codons, 1 indicates an intron inserted at the first base of a codon, 2 indicate an intron inserted at the second base of a codon. Figure was drawn using Gene Structure Display Server (GSDS) v2.0 [69].

## Slide 5
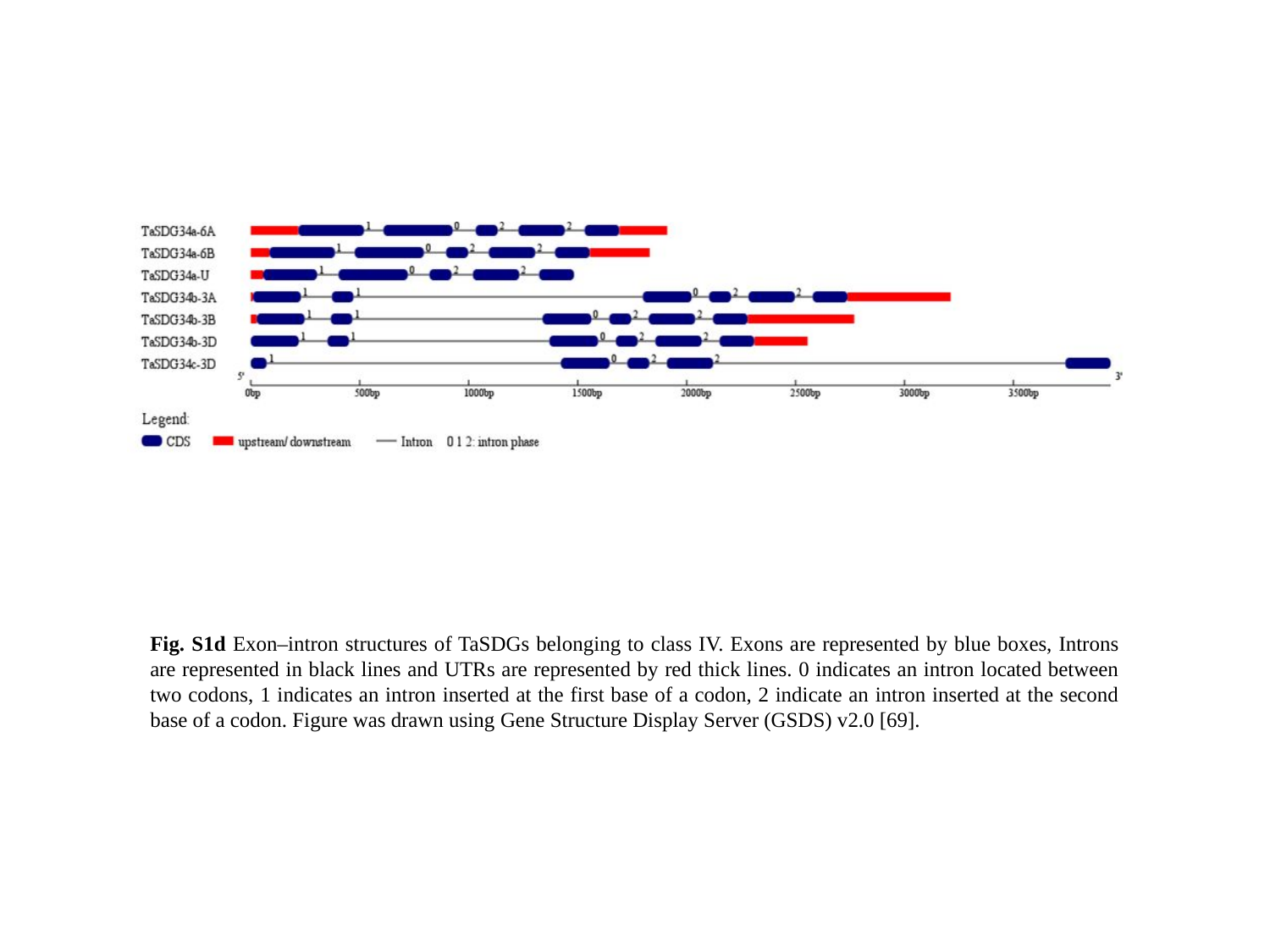

Fig. S1d Exon–intron structures of TaSDGs belonging to class IV. Exons are represented by blue boxes, Introns are represented in black lines and UTRs are represented by red thick lines. 0 indicates an intron located between two codons, 1 indicates an intron inserted at the first base of a codon, 2 indicate an intron inserted at the second base of a codon. Figure was drawn using Gene Structure Display Server (GSDS) v2.0 [69].

## Slide 6
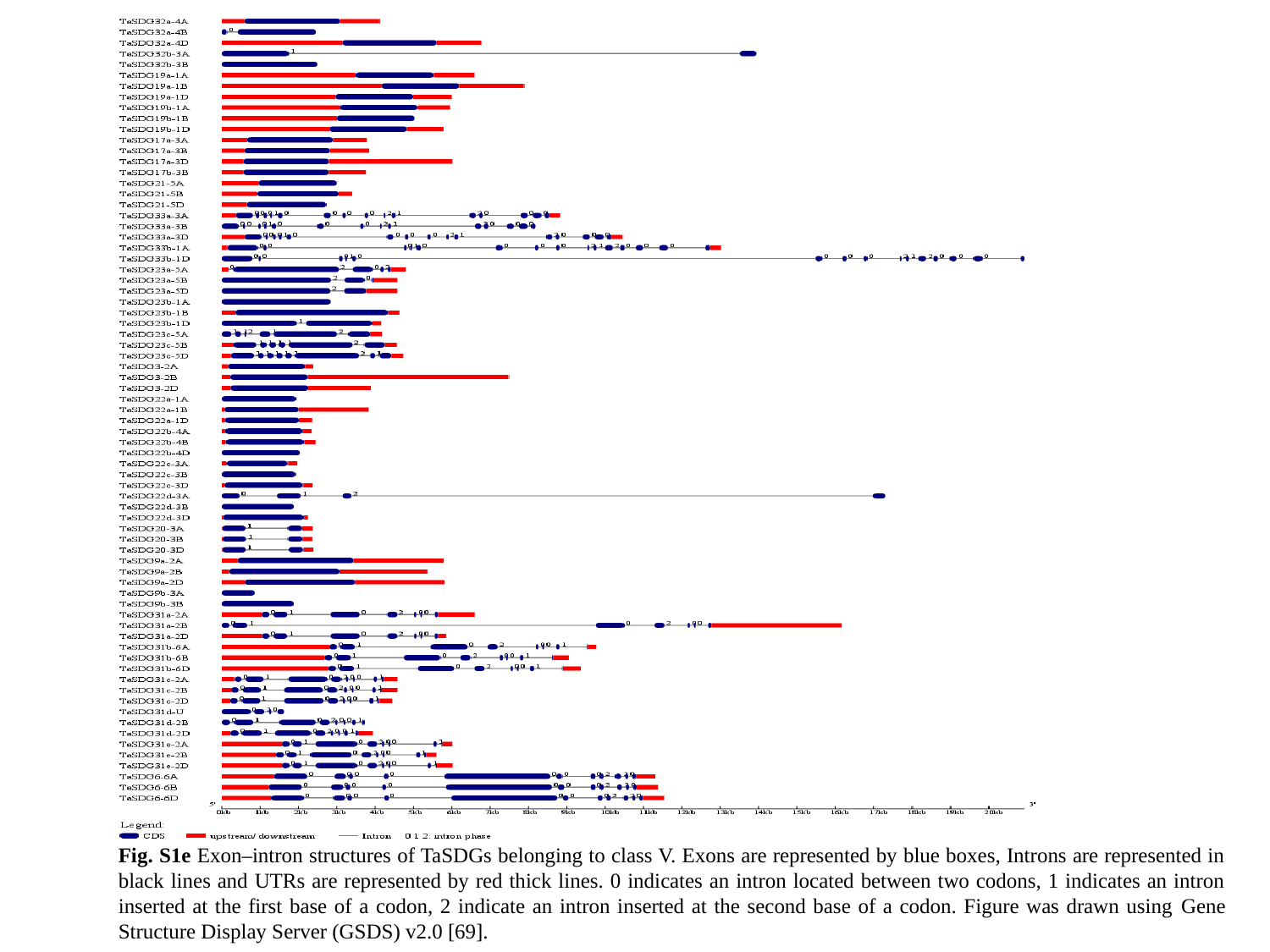

Fig. S1e Exon–intron structures of TaSDGs belonging to class V. Exons are represented by blue boxes, Introns are represented in black lines and UTRs are represented by red thick lines. 0 indicates an intron located between two codons, 1 indicates an intron inserted at the first base of a codon, 2 indicate an intron inserted at the second base of a codon. Figure was drawn using Gene Structure Display Server (GSDS) v2.0 [69].

## Slide 7
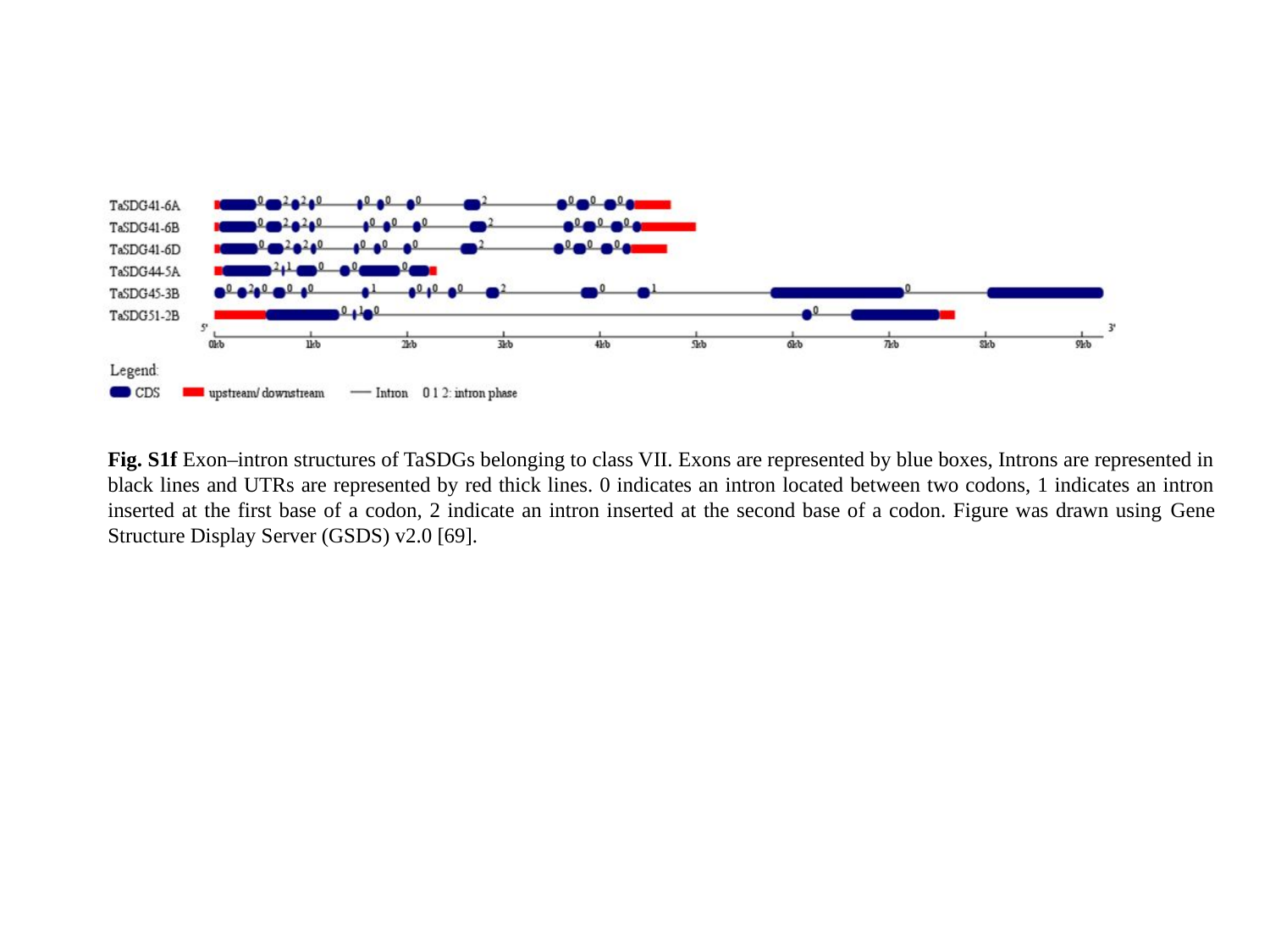

Fig. S1f Exon–intron structures of TaSDGs belonging to class VII. Exons are represented by blue boxes, Introns are represented in black lines and UTRs are represented by red thick lines. 0 indicates an intron located between two codons, 1 indicates an intron inserted at the first base of a codon, 2 indicate an intron inserted at the second base of a codon. Figure was drawn using Gene Structure Display Server (GSDS) v2.0 [69].

## Slide 8
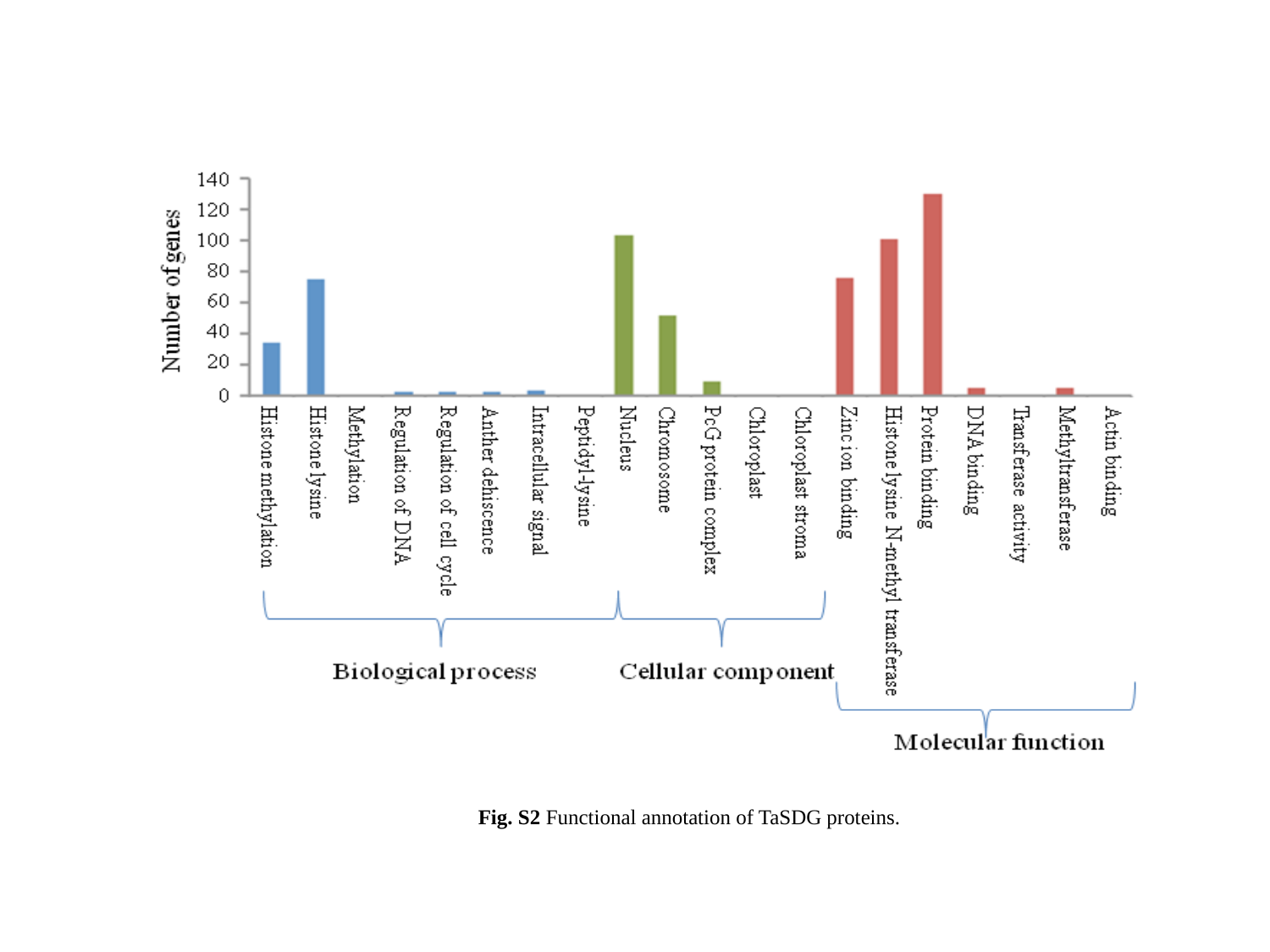

Fig. S2 Functional annotation of TaSDG proteins.
